# Zinc overload disrupts SoxR [2Fe–2S] clusters to drive redox-metallic crosstalk via SoxS-ZnuACB in *Escherichia coli*

**DOI:** 10.1016/j.redox.2026.104013

**Published:** 2026-01-08

**Authors:** Jie Feng, Feng Liang, Yongguang Zhou, Shihao Wen, Yue Chen, Binjie Ge, Wenjing Zhang, Jie Wang, Runyu Chen, Yin Zhang, Jianghui Li, Wu Wang, Guoqiang Tan

**Affiliations:** Laboratory of Molecular Medicine, Key Laboratory of Laboratory Medicine, Ministry of Education, School of Laboratory Medicine and Life Sciences, Wenzhou Medical University, Wenzhou, Zhejiang, 325035, China

**Keywords:** Iron sulfur cluster, Oxidative stress, Zinc homeostasis, SoxR, Redox regulation, Redox cycling reagent

## Abstract

Here, we demonstrate that excess zinc disrupts bacterial redox sensing by specifically disassembling the [2Fe–2S] cluster of SoxR – a master oxidative stress sensor in *Escherichia coli*. This impairment couples zinc overload to dysregulated oxidative defense, revealing a previously unrecognized metal-redox crosstalk mechanism. Using electron paramagnetic resonance (EPR) and UV–visible spectroscopy, we demonstrated that excess zinc specifically disrupts the assembly of the [2Fe–2S] cluster in redox-sensitive SoxR. Additionally, we assessed the expression levels of genes within this pathway using quantitative real-time PCR (qPCR) and quantified intracellular zinc and iron levels by inductively coupled plasma mass spectrometry (ICP-MS) to evaluate the roles of SoxS and the zinc uptake transporter ZnuACB in maintaining zinc homeostasis. Furthermore, we investigated the roles of SoxR, SoxS, and ZnuACB in bacterial zinc homeostasis through plate growth assays and gene knockout experiments. We establish that zinc excess disassembles SoxR [2Fe–2S] clusters as a molecular switch that dysregulates the SoxS-ZnuACB/SOD axis, converting zinc toxicity into oxidative vulnerability. This mechanistic insight exposes a bacterial Achilles' heel: targeting Fe–S cluster integrity disrupts redox-metal homeostasis, providing a strategy to combat antibiotic-resistant pathogens.

## 1 Introduction

Zinc is one of the most essential metal ions across all species, playing a critical role in the survival of pathogenic microorganisms [1]. It is the second most abundant metal in biological systems, following iron, and is a representative d-block metal. Although zinc is an essential element, excessive levels can be detrimental [2], as this metal can occupy binding sites intended for other biologically relevant metals. Consequently, maintaining intracellular zinc homeostasis is crucial for cellular survival. Zinc plays a pivotal role in cellular growth, development, homeostasis, transcription, cell division, oxidative stress response, apoptosis, and aging [3,4]. Zinc prevents the formation of free radicals and acts as an antioxidant, protecting thiol groups from oxidative damage. Beyond its catalytic and regulatory functions, zinc also serves as a structural component in numerous proteins, including regulatory proteins and transcription factors containing zinc-binding domains such as zinc fingers, loops, and LIM domains.

Iron-sulfur (Fe–S) clusters are ancient and ubiquitous cofactors that are conserved across nearly all bacteria, plants, and mammals [5]. They play crucial roles in various biological processes, including cellular respiration, photosynthesis, energy metabolism, RNA modification, protein translation, DNA replication and repair, and gene expression regulation [[6], [7], [8]]. The assembly mechanisms of Fe–S clusters are most commonly studied using *E*. *coli* as a model organism. *E. coli* harbors two Fe–S cluster assembly systems: the ISC and SUF systems. The ISC system, encoded by the *iscSUA-hscBA-fdx-iscX* operon, primarily facilitates Fe–S cluster biogenesis, transfer, and assembly under normal physiological conditions. In contrast, the SUF system, encoded by the *sufASCDSE* operon, functions predominantly under oxidative stress or iron-limiting conditions [9,10]. At present, Fe–S proteins have been discovered in a myriad of biological pathways [11]. SoxR is a 17-kDa polypeptide that belongs to the MerR family of transcriptional activators. Its primary structure predicts an N-terminal helix-turn-helix motif, a feature that appears to confer sequence-specific DNA binding within the MerR family [12]. The [2Fe–2S]-containing SoxR protein functions as a transcription factor that regulates the *soxRS* oxidative stress response in *E. coli*. In *E. coli*, SoxR directly regulates the expression of *soxS*, a transcription factor that promotes the production of various antioxidant proteins, including superoxide dismutase (SOD) [13] and the outer membrane drug efflux pump TolC [12]. Apo-SoxR, the iron-free form of the protein, remains dimeric and retains its ability to bind target DNA at the *soxS* promoter (K_D ∼10^−10^ M) [14]. Similarly, mutation of any cysteine residue in SoxR results in a stable dimeric protein that lacks a detectable [2Fe–2S] cluster yet retains strong DNA-binding affinity [15]. Thus, while the [2Fe–2S] cluster is not required to maintain the structural integrity of SoxR or its DNA-binding capacity, it is essential for activating *soxS* transcription. In the resting state, SoxR is constitutively bound to the *soxS* promoter, and its activity is governed by the redox state of its [2Fe–2S] cluster rather than by DNA binding. The reduced form [2Fe–2S]^1+^ is transcriptionally inactive, whereas oxidation to the [2Fe–2S]^2+^ state triggers a conformational change that enhances RNA polymerase recruitment and activates *soxS* transcription [16]. Apo-SoxR, despite remaining DNA-bound, cannot induce transcription. This redox-dependent switching mechanism allows SoxR to act as a sensor and signal transducer of oxidative stress in *E. coli*. SoxR senses oxidative stress through the oxidation state of its [2Fe–2S] cluster and regulates *soxS* transcription via redox-dependent conformational changes [17,18].

**Scheme 1:**
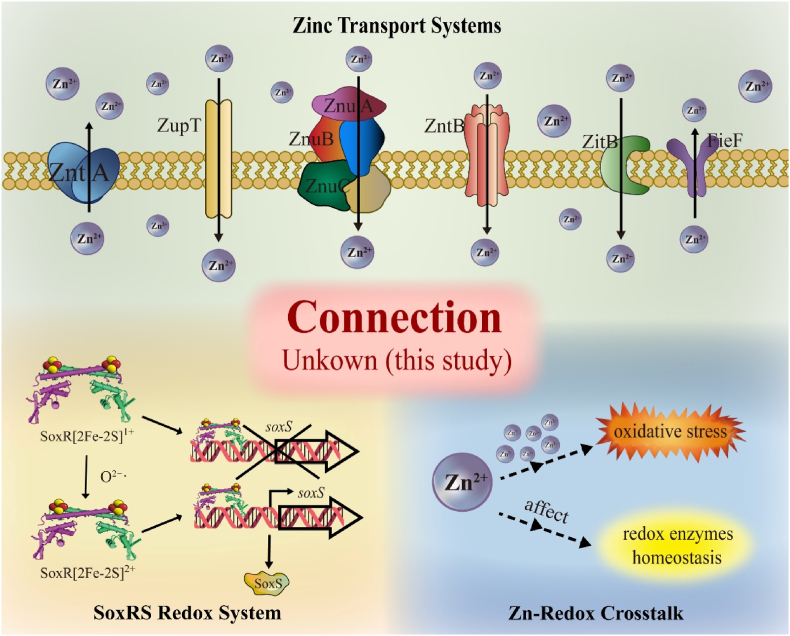
**Schematic overview of zinc transport, zinc–redox interactions, and the SoxR/SoxS regulatory system in *Escherichia coli*.** The upper panel illustrates the major zinc transport systems, including the high-affinity importer ZnuABC, the low-affinity transporter ZupT, and zinc efflux systems such as ZntA, ZitB, and FieF, which together maintain intracellular zinc homeostasis. The lower left panel depicts the SoxR–SoxS redox signaling module, in which SoxR constitutively binds the *soxS* promoter and senses oxidative stress through the redox state of its [2Fe–2S] cluster. Oxidation of the [2Fe–2S]^1+^ cluster to the [2Fe–2S]^2+^ state activates *soxS* transcription, leading to SoxS-dependent regulation of downstream genes. The lower right panel illustrates the reported association between zinc ions, oxidative stress, and redox enzyme homeostasis, in which zinc is known to influence redox balance through indirect and potentially multi-step mechanisms that remain incompletely defined. The central shaded region highlights the regulatory connection between zinc transport/homeostasis and the SoxR/SoxS redox system that remains unresolved prior to this study and is addressed in the present work.

Numerous types of transporters involved in intracellular zinc homeostasis have been identified in bacteria. Their abundance is transcriptionally regulated, typically mediated by zinc-dependent transcription factors. The flux of zinc depends on the number and type of transporters at specific cellular locations, as well as the amount of zinc they can transport [19]. The most critical components include zinc sensor proteins, membrane-bound transporters, and intracellular zinc-binding proteins. Zinc efflux and uptake in bacteria are primarily mediated by ATP-binding cassette (ABC) transporters, P-type ATPases, cation diffusion facilitators (CDFs), and the resistance-nodulation-division (RND) protein family [20]. The key bacterial sensors involved in zinc homeostasis include Zur (a member of the Fur family) and AdcR (of the MarR family), which regulate metal uptake; ZntR (of the MerR family), which controls metal efflux; and SmtB (of the ArsR family), which is responsible for metal efflux and storage [21]. The ZnuABC transporter, negatively regulated by Zur, is one of the most widely distributed high-affinity zinc uptake systems in Gram-negative bacteria. It facilitates the transport of zinc ions from the periplasmic space into the cytoplasm, leading to zinc accumulation within bacterial cells [22]. This system consists of the periplasmic zinc-binding protein ZnuA, the transmembrane permease ZnuB, and the ATPase ZnuC [23]. The regulatory mechanisms governing zinc homeostasis remain incompletely understood (Scheme 1). The dynamic equilibrium of intracellular zinc is orchestrated by multiple transporters and regulatory factors.

In previous studies, we reported that excessive intracellular copper can effectively block the biosynthesis of iron-sulfur clusters in *Escherichia coli* under both aerobic [24] and anaerobic [25] conditions. Furthermore, we found that zinc has a similar effect; excess zinc may target IscU, IscA, and Fdx in *E. coli*, thereby affecting its biosynthesis [26]. Here, to investigate the zinc homeostasis in *E. coli* and the molecular mechanisms by which zinc affects iron-sulfur cluster biosynthesis, we used electron paramagnetic resonance (EPR) and characteristic peak analysis of iron-sulfur clusters to confirm the specific interaction of zinc with SoxR. We also employed quantitative PCR (qPCR) to assess the expression levels of pathway-related genes, and inductively coupled plasma mass spectrometry (ICP-MS) to measure the total zinc and iron contents in the MC4100 strain and the iscU/iscA/fdx triple mutants. Additionally, we purified the relevant proteins to elucidate the mechanisms of zinc homeostasis in *E. coli*. Our findings indicate that *Escherichia coli* maintains its zinc homeostasis and antioxidant activity by regulating the SoxR-SoxS system, which in turn affects the expression of downstream genes, including *znuACB* and SOD.

## 2 Results

**Fig. 1:**
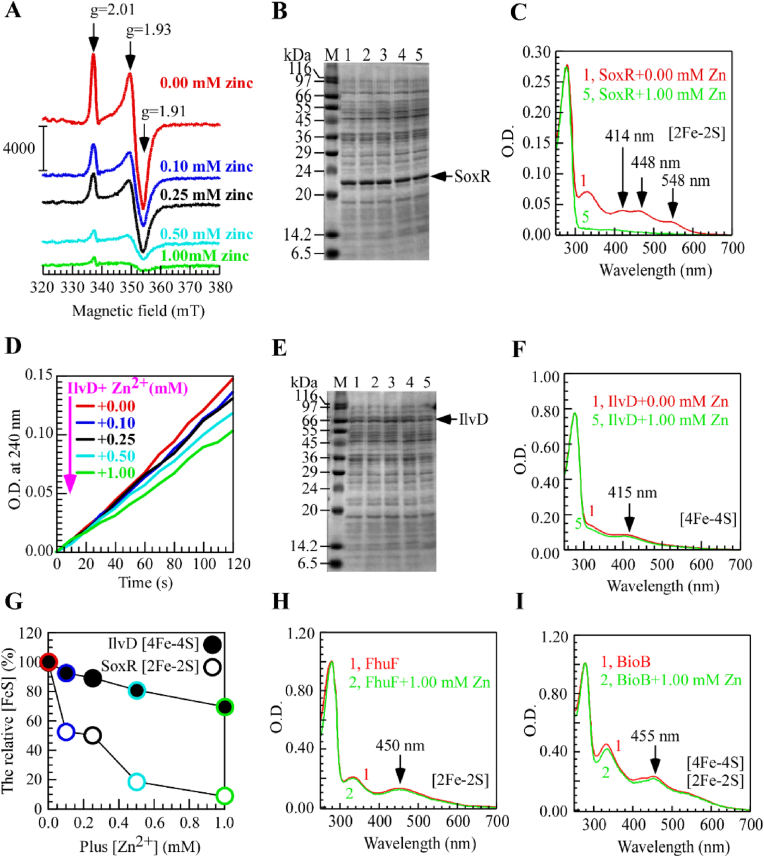
**Zinc specifically inhibits the assembly of the [2Fe–2S] cluster on SoxR in SoxR-overexpressing *E.coli* strains.** (**A**). Whole-cell electron paramagnetic resonance (EPR) analysis of SoxR-overexpressing *E. coli* cells of MC4100 cultured with zinc supplementation at concentrations of 0.10 mM, 0.25 mM, 0.50 mM, and 1.00 mM. The EPR spectra of all protein samples were baseline-corrected using buffer-only controls. (**B**). SDS-PAGE analysis of whole-cell assessing the effect of zinc on recombinant SoxR expression in *E. coli* cells of MC4100. Lanes 1–5 correspond to the gradient zinc concentrations in (A). (**C**). UV–visible absorption spectra of SoxR purified from *E. coli* cells of MC4100, comparing conditions with no added zinc in LB medium (spectrum 1) and with 1 mM ZnSO_4_ supplementation (spectrum 5). Spectrum 1 and 5 correspond to those in Figures A and B. (**D**). Enzyme activity assay of IlvD. Optical density at 240 nm was used to monitor the kinetic changes of dihydroxy-acid dehydratase (IlvD) activity in cell lysates upon zinc addition. The relationship between enzyme activity and reaction time was plotted. (**E**). SDS-PAGE analysis of whole-cell evaluating the effect of zinc on recombinant IlvD expression in *E. coli* cells of MC4100. (**F**). UV–visible absorption spectra of IlvD purified from *E. coli* cells of MC4100, comparing conditions with no added zinc in LB medium (spectrum 1) and with 1 mM ZnSO_4_ supplementation (spectrum 5). (**G**). Comparison of Fe–S cluster stability in IlvD (solid circles) and SoxR (open circles) as a function of increasing zinc concentration (0–1 mM). The relative Fe–S cluster content reflects the extent of Fe–S disruption caused by elevated zinc levels, evaluated based on the intensity of the reduced SoxR EPR signal and the relative IlvD enzymatic activity. (**H–I**). UV–visible absorption spectra of FhuF (H) and BioB (I) purified from *E. coli* cells of MC4100, comparing conditions with no added zinc in LB medium (spectrum 1) and with 1 mM ZnSO_4_ supplementation (spectrum 5). All experiments were performed at least three times, and the most representative results are shown.

**Zinc specifically inhibits the assembly of the [2Fe–2S] cluster on SoxR in SoxR-overexpressing *E.coli* strains.**–In our previous study, we reported that zinc overload in *E*. *coli* zntA zntR mutant cells inhibits iron-sulfur cluster biogenesis. To investigate the underlying mechanism, we hypothesized that this zinc overload might impair the iron-sulfur cluster biogenesis of the SoxR. EPR spectroscopy revealed that as the zinc ion concentration increased, the characteristic [2Fe–2S]^2+^ signal of the SoxR in *E*. *coli* MC4100 overexpressing SoxR strain gradually diminished (Fig. 1A). This phenomenon suggests that zinc ions may inhibit the assembly process or structural stability of the [2Fe–2S] cluster in SoxR. Whole-cell electrophoresis (Fig. 1B) demonstrated that zinc ions did not affect SoxR protein expression. However, EPR spectroscopy revealed signals corresponding to reduced [2Fe–2S]^+^ clusters. UV–Vis absorption spectra revealed that in the presence of 1 mM zinc ions, the characteristic absorption peak of the SoxR [2Fe–2S] cluster was significantly reduced compared to the control without zinc (Fig. 1C), further confirming the detrimental effect of zinc ions on the [2Fe–2S] cluster in SoxR. To explore the effect of zinc ions on other Fe–S–containing proteins, we examined IlvD, a [4Fe–4S] enzyme involved in branched-chain amino acid biosynthesis; FhuF, a [2Fe–2S] reductase involved in iron uptake; and BioB, a biotin synthase containing both [2Fe–2S] and [4Fe–4S] clusters. The results showed no significant changes in IlvD at different zinc ion concentrations (Fig. 1D), indicating that zinc ions have no significant effect on IlvD. Whole-cell electrophoresis (Fig. 1E) and UV–Vis absorption spectra (Fig. 1F) of IlvD were consistent, both showing no significant effect of zinc ions on IlvD protein. The analysis of Fe–S cluster stability in SoxR and IlvD (Fig. 1G) clearly demonstrated the zinc concentration–dependent disruption of the SoxR [2Fe–2S] cluster, with no apparent effect on IlvD. Additionally, UV–Vis absorption spectra of FhuF and BioB (Fig. 1H–I) were performed, yielding results similar to IlvD, with no significant changes induced by zinc ions. Based on these results, we conclude that in soxR-overexpressing *E. coli* strains, excess zinc ions specifically inhibit the assembly of the SoxR [2Fe–2S] cluster. This inhibitory effect was not observed in IlvD protein containing the [4Fe–4S] cluster, FhuF with the [2Fe–2S] cluster, or BioB protein containing both [2Fe–2S] and [4Fe–4S] clusters, indicating that zinc ions specifically affect the SoxR. These findings reveal the role of zinc ions in the fine-tuning of specific iron-sulfur protein functions, particularly the key role of SoxR in bacterial zinc homeostasis. In contrast, their impact on other iron-sulfur proteins is limited, providing new insights into how zinc ions influence bacterial oxidative stress responses and zinc homeostasis.

**Zinc disrupts the [2Fe–2S] cluster binding site in SoxR and affects protein stability in *E. coli* cells and *in vitro*.**–In *E. coli*, excess zinc effectively inhibits the function of SoxR, an iron–sulfur protein. In general, zinc affects iron–sulfur clusters via two mechanisms. First, zinc directly attacks the iron–sulfur cluster bound to a protein, competitively displacing the cluster and converting the protein to its apo form. Second, zinc can impede the synthesis of iron–sulfur clusters, resulting in proteins that, once translated, fail to incorporate the cluster and thus do not mature properly. Previous experiments (Fig. 1) demonstrated that zinc specifically inhibits the *in vivo* assembly of SoxR via the IscA/IscU/Fdx system. Based on these findings, we further investigated whether zinc ions affect SoxR function even after its assembly is complete.

**Fig. 2:**
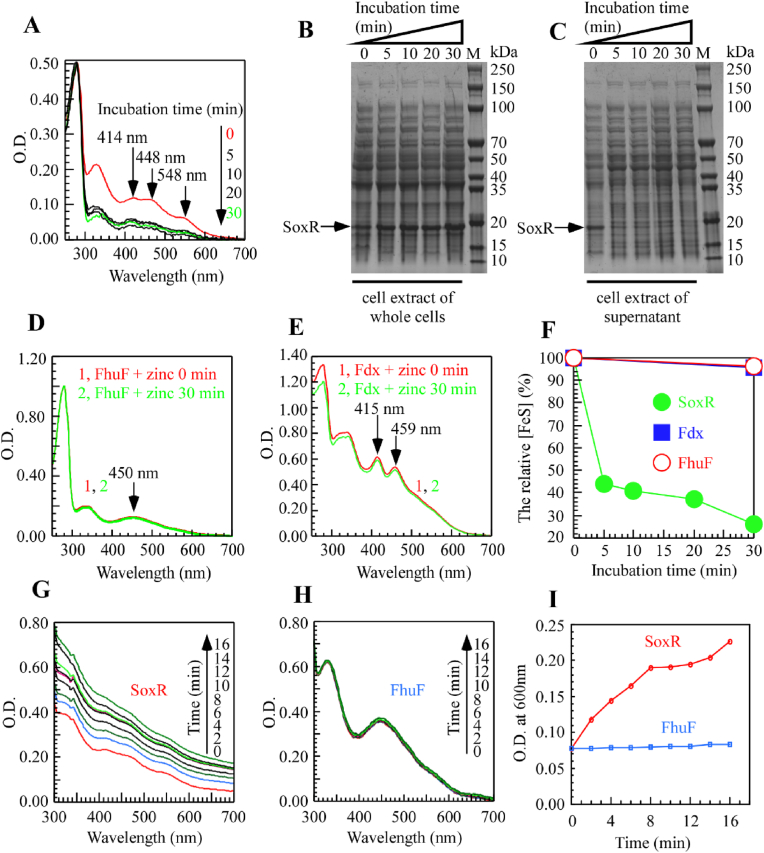
**Zinc disrupts the [2Fe–2S] cluster binding site in SoxR and affects protein stability in *E. coli* cells and *in vitro*.** (**A-C**). Kinetic analysis of the effect of zinc on the assembled [2Fe–2S] cluster in SoxR. MC4100 cells harboring the pBAD-*soxR* plasmid were induced with l-arabinose for 4 h. After induction, the culture medium was replaced, and chloramphenicol was added to inhibit new protein synthesis, followed by the addition of 1 mM ZnSO_4_. Samples were collected at 0, 5, 10, 20, and 30 min for further analysis, including UV–visible absorption spectroscopy after protein purification (A), whole-cell electrophoresis (B), and SDS-PAGE analysis of the supernatant after cell disruption (C). (**D-E**). UV–visible absorption spectra of FhuF (D) and Fdx (E) purified from wild-type *E. coli* cells of MC4100. The effect of adding 1 mM ZnSO_4_ post-induction in LB medium was analyzed. Chloramphenicol was included in all protein production conditions to inhibit new protein synthesis before zinc treatment. The kinetic changes in Fe–S cluster characteristic absorption peaks were monitored at selected time points (only 0 and 30 min were retained), following a procedure similar to that in panel A. (**F**). Time-dependent relative changes in Fe–S clusters in SoxR (solid circles), FhuF (solid squares), and Fdx (open circles) upon the addition of 1 mM ZnSO_4_ post-induction. (**G-H**). UV–visible absorption spectra of purified SoxR (G) and FhuF (H) incubated with 15 μM ZnSO_4_*in vitro* for 0–16 min. (**I**). Time-dependent absorbance changes of SoxR (red) and FhuF (blue) at 600 nm, the final concentration of the protein was normalized to 90 μM. All experiments were performed at least three times, and the most representative results are shown.

As shown in Fig. 2A, upon the addition of 1 mM ZnSO_4_ to LB medium, the characteristic absorption peaks of the [2Fe–2S] cluster in SoxR—expressed and purified from wild-type MC4100—were markedly reduced. This phenomenon persisted even when ZnSO_4_ was added after SoxR had fully matured. Within 5–30 min, the SoxR iron–sulfur cluster was nearly completely lost, and its spectral profile resembled that observed immediately after zinc addition, indicating that zinc's effect on the SoxR cluster is independent of the timing of its addition. As a control, the iron–sulfur cluster binding peaks of FhuF and Fdx remained essentially unchanged under the same conditions (Fig. 2D and E), suggesting that 1 mM zinc does not perturb the already assembled clusters in FhuF and Fdx but specifically affects the SoxR cluster. This finding implies that zinc ions may competitively bind to the metal sites on SoxR. Furthermore, time-course analysis (Fig. 2F) confirmed that the specific absorption peak of the SoxR [2Fe–2S] cluster gradually decreased over time, whereas those of FhuF and Fdx remained largely unchanged.

Further whole-cell electrophoresis analysis (Fig. 2B) revealed that, after SoxR iron–sulfur cluster assembly, the addition of 1 mM ZnSO_4_ resulted in a gradual weakening of the SoxR electrophoretic band over time, indicating a transition from a soluble to an insoluble state (Fig. 2C). This change was not observed in untreated *soxR*, suggesting that zinc addition induces structural alterations and reduces SoxR solubility. Since SoxR assembled *in vivo* was affected by zinc, we conducted *in vitro* experiments. To distinguish whether zinc affects SoxR during Fe–S cluster biogenesis or after cluster assembly, we treated cells with chloramphenicol to inhibit new protein synthesis. This approach allowed us to specifically examine the stability of preassembled SoxR [2Fe–2S] clusters upon zinc exposure. Upon adding 15 μM ZnSO_4_ to purified SoxR and FhuF proteins (each at a final concentration of 90 μM), scanning the 300–700 nm wavelength range revealed that optical density increased over time, and protein turbidity progressively intensified (Fig. 2G). This suggests that SoxR may react with zinc, leading to precipitation and an almost complete loss of the iron–sulfur absorption peak. In contrast, the control protein FhuF did not exhibit these changes (Fig. 2H). Furthermore, Fig. 2I shows that in the presence of 15 μM ZnSO_4_, the OD600 value for SoxR increased gradually over time, whereas FhuF remained largely unchanged. This observation is consistent with *in vivo* experiments, where zinc induced SoxR precipitation and iron–sulfur cluster loss, as evidenced by the UV–visible spectral changes in Fig. 2A.

In summary, whether prior to or following assembly or *in vitro*, high concentrations of zinc affect SoxR. Zinc reduces SoxR solubility and weakens its iron–sulfur absorption peak, causing a substantial portion of SoxR—devoid of its iron–sulfur cluster—to precipitate. It is also possible that zinc ions compete with iron for binding to cysteine residues within the cluster-binding motifs, thereby preventing the correct assembly of the Fe–S cluster and leading to SoxR inactivation. This substitution hypothesis is consistent with the observed decrease in the EPR signal and the instability of SoxR under high zinc conditions (Fig. 1A). As a result, SoxR loses its ability to respond to specific oxidative stresses, such as superoxide recognition.

**Fig. 3:**
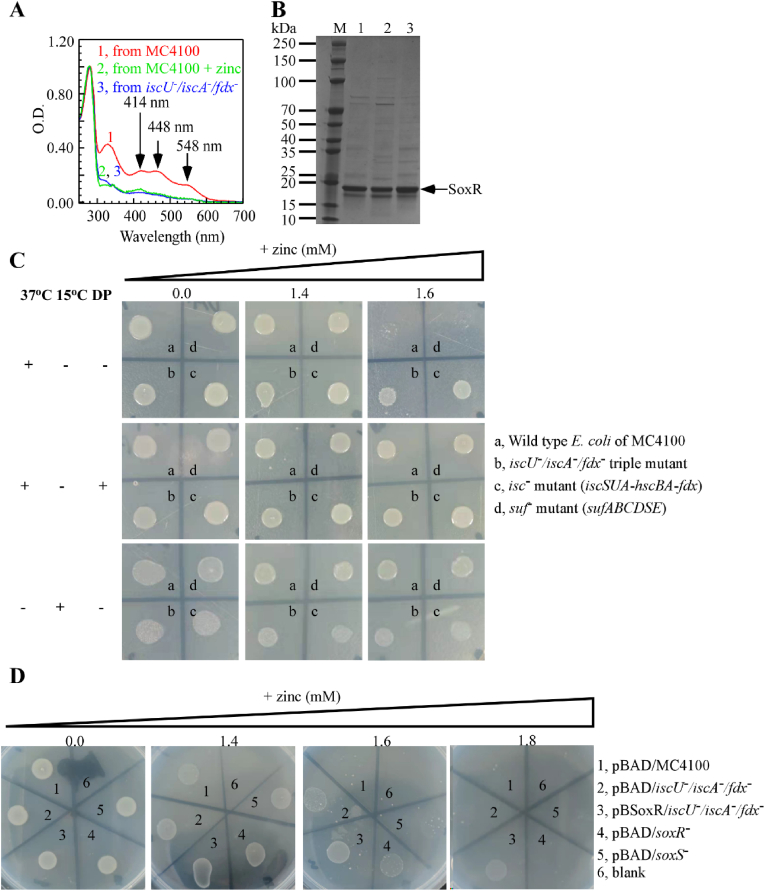
**Impaired iron–sulfur cluster assembly in SoxR confer enhanced tolerance to high external zinc environments in SoxR-overexpressing *E.coli* strains.** (**A**). UV–visible absorption spectra of recombinant SoxR purified from *E. coli* cells of MC4100 grown under aerobic conditions. Spectra compare SoxR purified from cells cultured in LB medium without ZnSO_4_ (spectrum 1), with 1 mM ZnSO_4_ supplementation (spectrum 2), and from the *iscU*^*-*^*/iscA*^*-*^*/fdx*^*-*^ mutant strain (spectrum 3). (**B**). SDS-PAGE analysis of the corresponding purified proteins. Lanes 1–3 correspond to spectra 1–3 in (A). (**C**). Zinc sensitivity analysis of Fe–S cluster assembly-deficient strains. Under aerobic conditions, wild-type *E. coli* cells of MC4100 (a), *iscU*^*-*^*/iscA*^*-*^*/fdx*^*-*^ triple mutant (b), *isc*^*-*^ (*iscSUA-hscBA-fdx-*) mutant (c), and *suf*^*-*^ (*sufABCDSE-*) mutant (d) were cultured on LB agar supplemented with 0.0 mM, 1.4 mM, or 1.6 mM ZnSO_4_ for 24 h. Parallel conditions included treatment with 200 μM iron chelator 2,2′-dipyridine (DP) or incubation at 15 °C. (**D**). Zinc sensitivity analysis of knockout strains. Under aerobic conditions, *E. coli* strains carrying different plasmid constructs—pBAD/MC4100 [1], pBAD/*iscU*^*-*^*/iscA*^*-*^*/fdx*^*-*^ [2], pBSoxR/*iscU*^*-*^*/iscA*^*-*^*/fdx*^*-*^ [3], pBAD/*soxR*^*-*^ [4], and pBAD/*soxS*^*-*^ [5]—were inoculated on LB agar plates containing 0 mM, 1.4 mM, 1.6 mM or 1.8 mM ZnSO_4_ and incubated for 24 h. All experiments were performed at least three times, and the most representative results are shown.

**Impaired iron–sulfur cluster assembly in SoxR confer enhanced tolerance to high external zinc environments in SoxR-overexpressing *E.coli* strains.**–To elucidate the mechanistic contributions of SoxR, we investigated the effects of zinc ions on the function of iron–sulfur proteins in *E. coli* and their role in zinc tolerance. UV visible absorption spectroscopy of the SoxR protein purified from *E. coli* MC4100 overexpressing *soxR* showed that the characteristic [2Fe–2S] absorption peaks at 414, 448, and 548 nm were markedly reduced upon addition of 1 mM Zinc ions, indicating that zinc interferes with Fe–S cluster stability in *E. coli* (Fig. 3A). Furthermore, analyses showed that the characteristic peaks of the SoxR [2Fe–2S] cluster in the *iscU*^−^/*iscA*^−^/*fdx*^−^ triple mutant were similar to those observed in the wild-type strain treated with 1 mM zinc, suggesting that strains with impaired iron–sulfur cluster assembly exhibit phenotypes analogous to zinc-treated wild-type cells (Fig. 3A and B).

Previous experiments demonstrated that the characteristic peaks of iron–sulfur clusters in wild-type MC4100 and in the *iscU*^−^/*iscA*^−^/*fdx*^−^ knockout were suppressed under high zinc stress [26]. Therefore, we employed the membrane-permeable iron chelator 2,2′-dipyridine (DP) and low-temperature cultivation (Fig. 3C) to induce impaired iron–sulfur cluster assembly [27], thereby investigating whether such assembly defects—induced by alternative methods—also affect *E. coli* resistance to zinc ions. At 37 °C without DP, the results were consistent with earlier findings: the *iscU*^−^/*iscA*^−^/*fdx*^−^ and *isc*^−^ strains exhibited enhanced zinc resistance, whereas the control strains, MC4100 and *suf*^−^, displayed inhibited growth on LB plates supplemented with 1.6 mM ZnSO_4_. Upon the addition of DP, iron–sulfur cluster assembly in MC4100 and *suf*^−^ was similarly impaired, and plate assays revealed that the growth of MC4100 and *suf*^−^ under 1.6 mM ZnSO_4_ was comparable to that of the *iscU*^−^/*iscA*^−^/*fdx*^−^ and *isc*^−^ strains, indicating increased zinc resistance. Under low-temperature conditions (15 °C), iron–sulfur cluster assembly was also inhibited, yielding results analogous to those obtained at 37 °C with DP [27]; that is, at low temperature, the growth of MC4100 and *suf*^−^ on 1.6 mM ZnSO_4_ was similar to that of the *iscU*^−^/*iscA*^−^/*fdx*^−^ and *isc*^−^ strains. Thus, both DP and low temperature, by impairing ISC system–mediated iron–sulfur cluster assembly, confer resistance to zinc toxicity under high zinc concentrations.

To explore whether zinc exerts a specific effect on SoxR—which activates transcription of the *soxS* gene and functions as an oxy-radical sensor—we performed plate assays using *soxR*^*-*^, *soxS*^*-*^, and the triple knockout strains. The results showed that at 1.6 mM Zn, the *soxR*^−^, *soxS*^−^, and *iscU*^−^/*iscA*^−^/*fdx*^−^ knockout strains exhibited the same phenotype (Fig. 3D), namely, enhanced zinc tolerance. Moreover, we found that the *soxR* complemented *iscU*^*-*^*/iscA*^*-*^*/fdx*^*-*^ triple knockout strain exhibited greater resistance to zinc stress (Fig. 3D). This may be due to the formation of apo-SoxR upon exposure to high zinc levels, which tightly binds to the *soxS* promoter, inhibiting its transcription and consequently enhancing tolerance to high-zinc conditions. This finding is consistent with the putative roles of SoxR and SoxS in regulating zinc tolerance. In summary, we conclude that SoxR deletion and defects in iron–sulfur cluster assembly similarly enhance *E. coli* resistance to high external zinc levels.

**Fig. 4:**
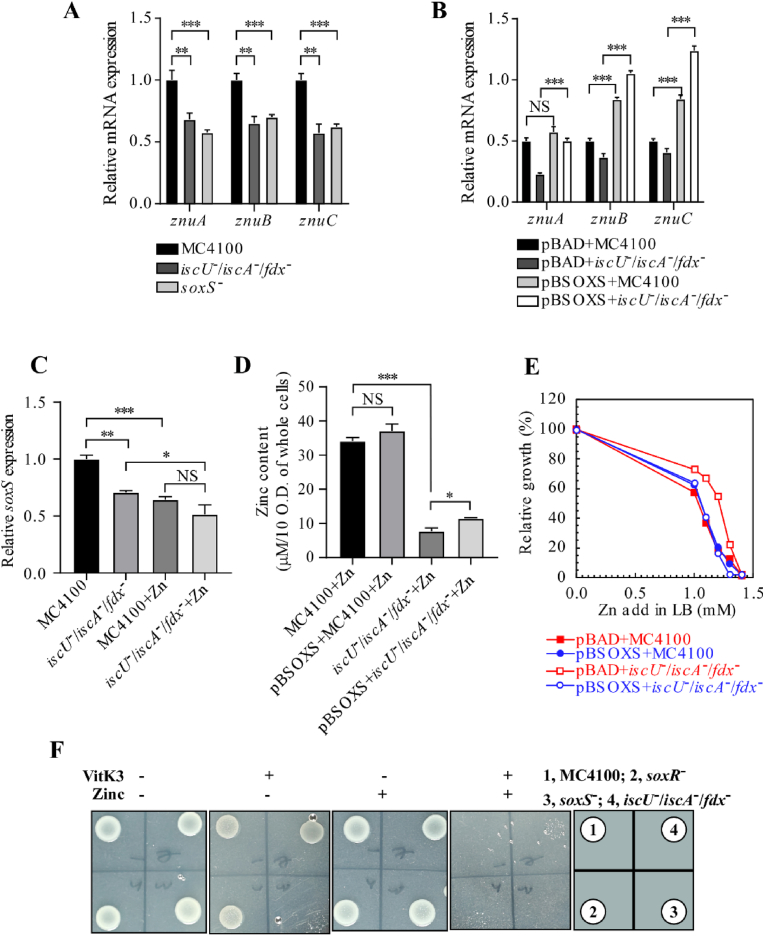
**The resistance of Fe–S cluster assembly-deficient bacteria to zinc is primarily mediated by SoxS and ZnuACB.** (**A**). Quantitative real-time PCR (qPCR) analysis of *znuACB* transcription levels in *E. coli* cells of MC4100, *iscU*^*-*^*/iscA*^*-*^*/fdx*^*-*^, and *soxS*^*-*^ strains. (**B**). qPCR analysis of *znuACB* transcription levels in *E. coli* cells of pBAD/MC4100, pBAD/*iscU*^*-*^*/iscA*^*-*^*/fdx*^*-*^, pBSoxS/MC4100, and pBSoxR/*iscU*^*-*^*/iscA*^*-*^*/fdx*^*-*^ strains after *soxS* complementation. (**C**). qPCR analysis of *soxS* transcription levels in *E. coli* cells of MC4100 and *iscU*^*-*^*/iscA*^*-*^*/fdx*^*-*^ strains, with or without 1.2 mM ZnSO_4_ in LB medium. (**D**). Total zinc content in *E. coli* cells of MC4100, pBSoxS/MC4100, *iscU*^*-*^*/iscA*^*-*^*/fdx*^*-*^, and pBSoxR/*iscU*^*-*^*/iscA*^*-*^*/fdx*^*-*^ strains cultured in LB medium supplemented with 1.2 mM ZnSO_4_, as determined by inductively coupled plasma mass spectrometry (ICP-MS). (**E**). Growth curves of pBAD + MC4100, pBAD-SoxS + MC4100, pBAD + *iscU*^*-*^*/iscA*^*-*^*/fdx*^*-*^, and pBAD-SoxS + *iscU*^*-*^*/iscA*^*-*^*/fdx*^*-*^ strains in LB medium supplemented with 0.0, 1.0, 1.1, 1.2, 1.3, or 1.4 mM ZnSO_4_. (**F**). Sensitivity assay of *E. coli* to combined zinc and redox cycling reagent vitamin K3. Under aerobic conditions, *E. coli* cells of MC4100 [1], *soxR*^*-*^ [2], *soxS*^*-*^ [3], and *iscU*^*-*^*/iscA*^*-*^*/fdx*^*-*^ [4] were inoculated on LB agar plates with or without 1 mM Zn^2+^ and 0.6 mM redox cycling reagent (VitK_3_) and incubated for 24 h. For (A–D), qPCR data were normalized to the internal reference gene *mdoG*. Data in (A–E) represent the mean ± standard deviation (SD) of three independent experiments. In (F), the most representative result from three independent replicates is shown. ∗*P < 0.05*, ∗∗*P < 0.01*, ∗∗∗*P < 0.001*; *ns* indicates no significant difference.

**The resistance of Fe–S cluster assembly-deficient bacteria to zinc is primarily mediated by SoxS and ZnuACB.**– As shown in Fig. 4A, the mRNA expression levels of *znuACB* in *soxS*^−^ and *iscU*^−^/*iscA*^−^/*fdx*^−^ knockout strains were comparable, both exhibiting a degree of downregulation. Given the similarity to previous observations, we hypothesized that SoxS may regulate *znuACB*. To test this, we overexpressed SoxS and examined *znuACB* mRNA expression in pBAD/MC4100 and *iscU*^−^/*iscA*^−^/*fdx*^−^ knockout strains transformed with pBAD-SoxS. The results (Fig. 4B) showed that overexpression of SoxS led to increased *znuACB* mRNA levels in both strains. These findings suggest that SoxS enhances the expression of *znuACB* by regulating its uptake transporter.

Based on this observation, we hypothesized that SoxS overexpression increases intracellular zinc levels in *E. coli*. To test this, we measured total intracellular zinc content using ICP-MS in MC4100 and *iscU*^−^/*iscA*^−^/*fdx*^−^ knockout strains grown in the presence of 1.2 mM ZnSO_4_. As shown in Fig. 4D, no significant difference was observed in intracellular zinc levels between MC4100 and pBAD-SoxS/MC4100. However, in pBAD-SoxS/*iscU*^−^/*iscA*^−^/*fdx*^−^ strains, intracellular zinc content was markedly elevated compared with *iscU*^−^/*iscA*^−^/*fdx*^−^ knockout strains. The increased zinc concentration subjected pBAD-SoxS/*iscU*^*-*^*/iscA*^*-*^*/fdx*^*-*^ knockout strains to zinc toxicity stress, which may be the primary cause of the phenotype observed in Fig. 4E. As shown in Fig. 4C, the mRNA expression level of *soxS* decreased in both *E. coli* cells of MC4100 and *iscU*^*-*^*/iscA*^*-*^*/fdx*^*-*^ knockout strains after the addition of 1.2 mM ZnSO_4_. Moreover, the *soxS* mRNA levels in *iscU*^*-*^*/iscA*^*-*^*/fdx*^*-*^ knockout strains were similar to those in MC4100 under 1.2 mM ZnSO_4_ treatment. Previous experiments confirmed that zinc disrupts the [2Fe–2S] cluster in SoxR, leading to the formation of apo-SoxR, which is incapable of activating its downstream target gene *soxS*, thereby resulting in reduced *soxS* mRNA expression (Fig. 1, Fig. 2). Similarly, the [2Fe–2S] cluster in SoxR purified from *iscU*^*-*^*/iscA*^*-*^*/fdx*^*-*^ knockout strains was also affected, leading to a comparable reduction in *soxS* mRNA expression.

We previously proposed that zinc ions specifically inhibit SoxR, which functions as a superoxide response regulator. SoxR activates the transcription of *soxS*, which in turn influences the activity of superoxide dismutase (SOD), thereby modulating bacterial oxidative stress resistance. To test this, we supplemented LB agar plates with the oxidant vitamin K3 (menadione) and 1 mM ZnSO_4_ (Fig. 4F). Vitamin K3, functioning as a redox cycling reagent, induces superoxide anion generation in *E*. *coli*. On zinc-free plates, the growth of *soxS*^−^ strains was inhibited in the presence of an oxidant, indicating that *soxS* knockout affects SOD activity and consequently impairs bacterial oxidative stress resistance. Upon the addition of 1 mM zinc, Zn-SoxR-SoxS signaling influenced SOD activity, and the growth differences among MC4100, *soxR*^−^, *iscU*^−^/*iscA*^−^/*fdx*^−^ knockout strains, and *soxS*^−^ strains on plates largely disappeared, supporting the validity of our proposed pathway. Moreover, we noticed that the combined use of zinc and vitamin K3 shows promising antibacterial potential. Therefore, we quantitatively analyzed the concentrations in Fig. 4F and demonstrated that bacterial growth was inhibited under lower stress conditions when both agents were used together (Supplemental Fig. 1). Next, we complemented MC4100 and *soxS*^−^ strains with the empty vector pBAD or pBAD-SoxS (Supplemental Fig. 2). On zinc-free LB plates, SoxS-complemented MC4100 and *soxS*^−^ strains exhibited greater resistance to oxidants than control strains. However, upon the addition of 0.6 mM Zn, SoxS activity was suppressed due to Zn-mediated inhibition of SoxR, yet SoxS complementation still conferred greater oxidative resistance compared with control strains. In conclusion, these findings confirm the role of SoxS in oxidative stress resistance and support the function of the Zn-SoxR-SoxS-SOD pathway in *E. coli*.

**Fig. 5:**
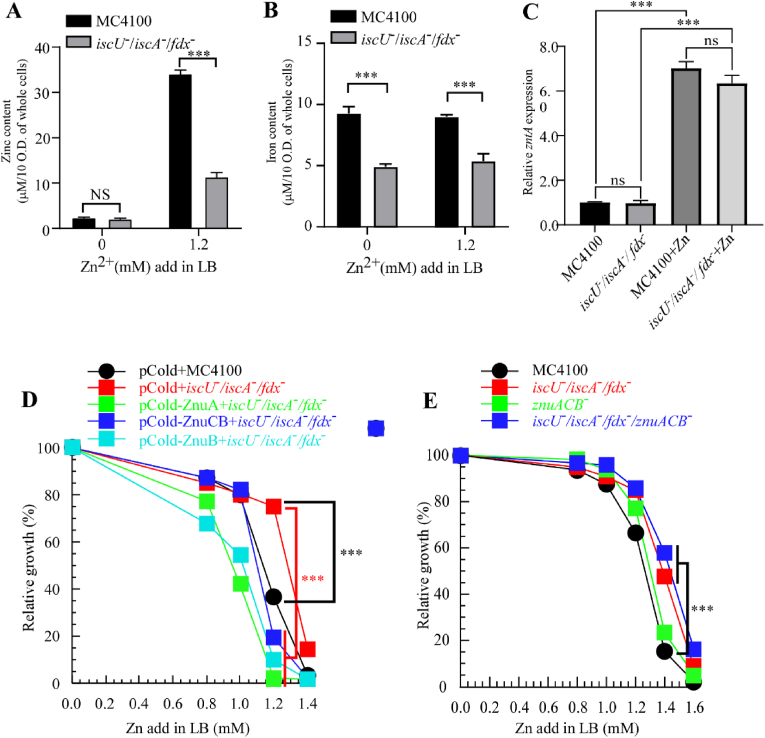
**Under high-zinc conditions, zinc tolerance in *E. coli* involves modulation of the zinc uptake transporter ZnuACB rather than the efflux system zntR/zntA.** (**A**). Total zinc content in *E. coli* cells of MC4100 and *iscU*^*-*^*/iscA*^*-*^*/fdx*^*-*^ strains cultured in LB medium with or without 1.2 mM ZnSO_4_, as determined by inductively coupled plasma mass spectrometry (ICP-MS). (**B**). Total iron content in *E. coli* cells of MC4100 and *iscU*^*-*^*/iscA*^*-*^*/fdx*^*-*^ strains cultured in LB medium with or without 1.2 mM ZnSO_4_, as determined by inductively coupled plasma mass spectrometry (ICP-MS). (**C**). Quantitative real-time PCR (qPCR) analysis of zntA transcription levels in *E. coli* cells of MC4100, iscU^−^/iscA^−^/fdx^−^, and both strains cultured with 1 mM ZnSO_4_. Data were normalized to the internal reference gene *mdoG*. (**D**). Growth curves of wild-type *E. coli* cells of MC4100, *iscU*^*-*^*/iscA*^*-*^*/fdx*^*-*^ triple mutants, and *iscU*^*-*^*/iscA*^*-*^*/fdx*^*-*^ strains complemented with pCold-*znuA*, pCold-*znuCB*, or pCold-*znuB*, grown in LB medium supplemented with 0.0, 0.8, 1.0, 1.2, or 1.4 mM ZnSO_4_. (**E**). Growth curves of wild-type *E. coli* cells of MC4100, *iscU*^*-*^*/iscA*^*-*^*/fdx*^*-*^ triple mutants, *znuACB*^−^, and *iscU*^*-*^*/iscA*^*-*^*/fdx*^*-*^/*znuACB*^−^ quadruple mutants strains, grown in LB medium supplemented with 0.0, 0.8, 1.0, 1.2, 1.4, or 1.6 mM ZnSO_4_. (All experiments were performed in triplicate. ∗*P < 0.05*, ∗∗*P < 0.01*, ∗∗∗*P < 0.001*; *ns* indicates no significant difference.)

**Under high-zinc conditions, zinc tolerance in *E. coli* involves modulation of the zinc uptake transporter ZnuACB rather than the efflux system zntR/zntA.**–Given that *soxS* transcriptionally regulates the zinc uptake system ZnuACB, we systematically investigated the functional contribution of the ZnuACB transporter to zinc homeostasis. In this study, as shown in Fig. 5A, ICP-MS analysis revealed that in the absence of exogenous zinc, intracellular zinc concentrations in both strains were comparable. However, upon the addition of 1.2 mM zinc, intracellular zinc levels in the *iscU*^−^/*iscA*^−^/*fdx*^−^ triple mutant strain were significantly lower than those in the wild type. This phenomenon was closely associated with the downregulation of the zinc uptake transporter *znuACB*, which functions downstream of SoxS. Fig. 5B shows that the triple mutant strain maintained a consistently lower intracellular iron concentration, regardless of zinc stress conditions. This reduction in iron levels likely resulted from *iscA* deletion, which impairs iron–sulfur cluster assembly and subsequently suppresses “chelatable iron pool” [28]. The observed reduction in intracellular zinc levels may be attributed to the downregulation of the zinc uptake transporter *znuACB* or the upregulation of zinc efflux transporters *zntR/zntA*. To further investigate this, we performed qPCR analysis of the zinc uptake transporter genes. The results showed that *znuACB* mRNA expression was lower in *iscU*^−^/*iscA*^−^/*fdx*^−^ knockout strains than in wild-type MC4100 (Fig. 4A), confirming transcriptional suppression of this gene in the triple mutant. In contrast, no significant difference was observed in the expression of the zinc efflux genes *zntR* and *zntA* (Fig. 5C). These findings suggest that the downregulation of *znuACB* may serve as a molecular mechanism by which bacteria adapt to high-zinc environments and enhance zinc tolerance.

Based on our previous hypothesis—that the defective Fe–S cluster assembly of SoxR influences downstream SoxS expression and thereby regulates the zinc uptake system ZnuACB—we introduced pCold-*ZnuA*, pCold-*ZnuCB*, and pCold-*ZnuB* into both the *iscU*^*-*^*/iscA*^*-*^*/fdx*^*-*^ knockout strain and the wild-type MC4100 strain (Fig. 5D; Supplemental Fig. 3A). All three complemented strains exhibited increased sensitivity to zinc. When 1.2 mM ZnSO_4_ was added to LB agar plates, a significant difference in growth was observed between the triple mutant and MC4100. Moreover, following znuACB complementation, all strains showed markedly reduced growth. Notably, at 1.4 mM ZnSO_4_, no visible single colonies were observed for any strain except the *iscU*^*-*^*/iscA*^*-*^*/fdx*^*-*^ triple knockout (Supplemental Fig. 3B). To further validate the role of ZnuACB in zinc homeostasis, we generated a ZnuACB deletion strain, as well as a quadruple knockout by additionally deleting *znuACB* in the *iscU*^*-*^*/iscA*^*-*^*/fdx*^*-*^ background (Fig. 5E). Both the *znuACB* single knockout and the quadruple mutant exhibited significantly enhanced zinc tolerance. These findings demonstrate that the zinc uptake system ZnuACB plays a crucial role in maintaining zinc homeostasis.

As shown in Supplemental Fig. 4, fumarase activity was measured following the introduction of *znuACB* into *fumC*^−^ strains. The results revealed a significant decrease in fumarase activity in *znuACB*-expressing strains. Since both fumarase A and B contain [4Fe–4S] cluster proteins, this reduction in activity suggests that *znuACB* expression disrupts [4Fe–4S] cluster assembly in *E. coli*. This impairment may result from excessive zinc uptake, which induces zinc toxicity and disrupts the function of iron–sulfur cluster assembly proteins (IscU, IscA, Fdx) [26]. These findings further support that the zinc uptake transporter *znuACB* plays a crucial role in conferring zinc tolerance in *iscU*^−^/*iscA*^−^/*fdx*^−^ knockout strains.

Collectively, the results in Fig. 4, Fig. 5 suggest that the *iscU*^−^/*iscA*^−^/*fdx*^−^ triple mutant reduces intracellular zinc accumulation and enhances zinc tolerance primarily by downregulating *znuACB* expression rather than upregulating *zntR/zntA*.

## 3 Discussion

**Fig. 6:**
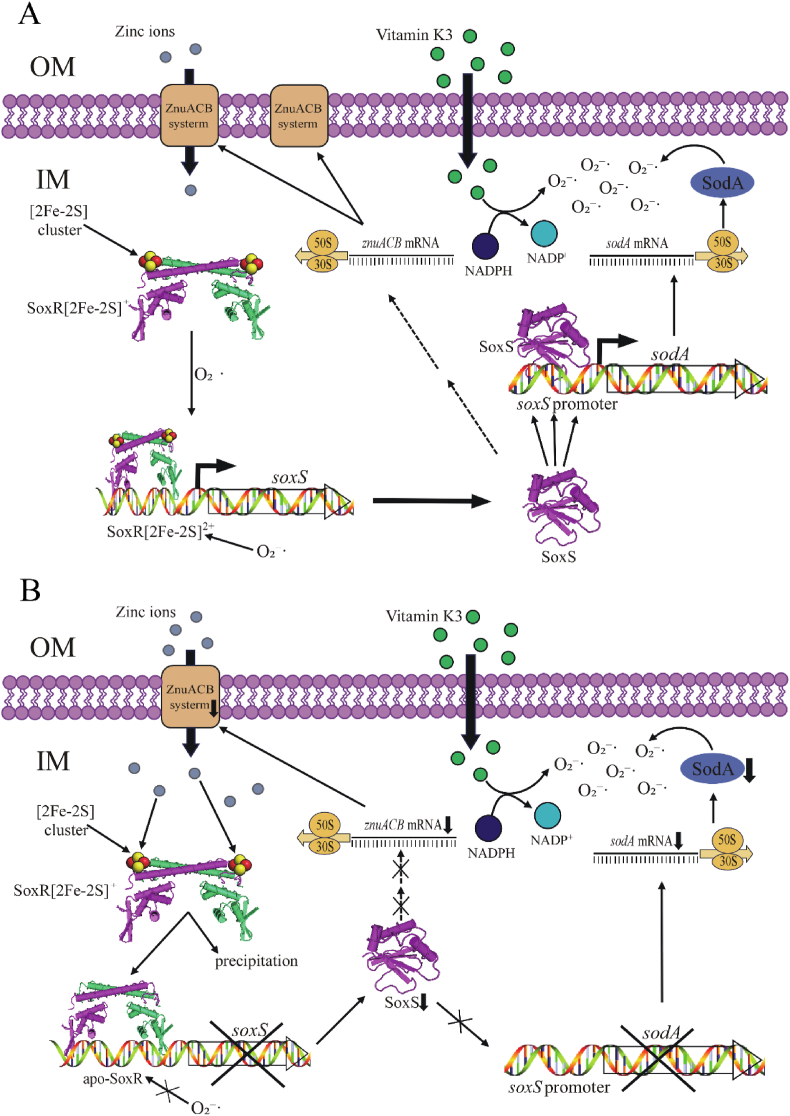
**Proposed mechanism of zinc homeostasis regulated by SoxR in *E. coli* cells.** Schematic representation of zinc homeostasis regulation under normal (**A**) and high-zinc (**B**) conditions in *E. coli*. When bacteria are exposed to excessive zinc in the external environment, zinc ions affect the [2Fe–2S] cluster of SoxR, leading to the formation of a largely apo-SoxR that tightly binds to the *soxS* promoter. This suppresses SoxS-mediated expression of the zinc uptake transporter *znuACB*, thereby reducing zinc uptake to maintain bacterial zinc homeostasis. On the other hand, the redox cycling reagent vitamin K_3_ generates reactive oxygen species, which, in combination with high zinc-induced *sodA* silencing, forms a novel antibacterial strategy.

In this study, we elucidated the molecular mechanism by which zinc influences both zinc homeostasis and oxidative stress responses in *E*. *coli* through the SoxR-SoxS regulatory axis. Using electron paramagnetic resonance (EPR) and UV–visible spectroscopy, we demonstrated that zinc ions specifically inhibit the assembly of the SoxR [2Fe–2S] cluster, thereby affecting its activity. Furthermore, *in vitro* experiments with purified SoxR protein revealed that zinc induces SoxR precipitation, leading to functional loss. These findings confirm the dual role of zinc in modulating SoxR both *in vivo* and *in vitro*. The reduction in SoxR activity further suppressed the expression of its downstream regulator SoxS [29,30], which in turn directly influenced the expression of the zinc transport system ZnuACB and superoxide dismutase (SOD) [31]. Under high-zinc conditions (Fig. 6B), *E. coli* downregulates *znuACB* expression via this regulatory axis to limit zinc uptake while modulating SOD expression to enhance oxidative stress resistance [32]. Additionally, we observed that the *iscU*^*-*^*/iscA*^*-*^*/fdx*^*-*^ mutant exhibited greater zinc tolerance. Moreover, inducing Fe–S cluster assembly defects using iron chelators and low-temperature treatments further enhanced zinc tolerance in *E. coli*, suggesting that impaired Fe–S cluster biogenesis may confer an adaptive advantage under high-zinc conditions. This study identifies a novel Zn-SoxR-SoxS-ZnuACB/SOD signaling pathway in *E. coli* (Fig. 6A), which plays a critical role in bacterial adaptation to high-zinc environments and oxidative stress regulation. These findings provide new insights into the mechanisms governing metal homeostasis in prokaryotes.

Our previous studies demonstrated that copper disrupts Fe–S cluster biogenesis under both aerobic and anaerobic conditions [24,25]. Similarly, zinc may regulate Fe–S cluster biosynthesis by targeting IscU, IscA, and ferredoxin, thereby influencing Fe–S cluster assembly in *E*. *coli*. [26]. In this study, we further investigated the regulatory mechanisms by which *E. coli* adapts to high extracellular zinc levels. Our preliminary experiments revealed that the mRNA expression level of the zinc uptake system *znuACB* was lower in the *iscU*^*-*^*/iscA*^*-*^*/fdx*^*-*^ knockout strain than in the MC4100 strain. We further observed that deletion of *znuACB* conferred increased zinc tolerance in bacteria, whereas complementation of *znuACB* restored zinc sensitivity. We also considered the low-affinity zinc uptake system ZupT. Our results indicated that its expression is not regulated by zinc levels (data not shown), a finding that is consistent with previous literature [33]. Additionally, we excluded the possibility that the zinc efflux transporters *zntA/zntR* were involved in this regulation. Based on these findings, we hypothesized that Fe–S cluster assembly defects enhance zinc resistance in the *iscU*^*-*^*/iscA*^*-*^*/fdx*^*-*^ mutant by downregulating *znuACB* expression. This reduction in *znuACB* expression likely decreases total intracellular zinc levels, thereby conferring resistance to zinc toxicity.

The zinc uptake regulator (Zur) belongs to the Fur superfamily of transcriptional regulators and is widely distributed among bacterial species [34,35]. When Zur forms a complex with Zn^2+^ and binds to conserved AT-rich sequences, it represses *znuACB* expression, a process essential for preventing excessive intracellular zinc accumulation. Previous studies have shown that SoxS, a key regulator of oxidative stress responses and antibiotic resistance, can activate the expression of the zinc uptake system *znuACB*. However, this activation appears to be indirect, as gel shift assays failed to detect SoxS binding to the *znuACB* promoter [36]. In addition, relavent studies also have demonstrated that ZinT functions as a zinc-buffering protein capable of transferring Zn^2+^ to ZnuA [37,38]. Furthermore, this metallochaperone facilitates metal recruitment under zinc-depleted conditions through regulation by the *soxS* gene [39]. Given that SoxS is unlikely to directly interact with the *znuACB* promoter and that *znuACB* is negatively regulated by Zur [40], we hypothesize that SoxS indirectly regulates *znuACB* by repressing Zur or ZinT. However, the precise mechanism remains to be elucidated.

The SoxR protein contains a [2Fe–2S] cluster that is essential for its transcriptional activation function [41]. This cluster acts as a redox sensor for superoxide detection [42]. Upon reduction of the [2Fe–2S]^2+^ cluster, SoxR reversibly loses its transcriptional activity while retaining its DNA-binding affinity. In *E*. *coli*, the primary target gene of SoxR is *soxS*, which SoxR activates at the transcriptional level [29,43,44]. Based on this information, we hypothesized that SoxR might be closely linked to zinc homeostasis in *E. coli*. To test this hypothesis, we purified recombinant SoxR protein from *E. coli* MC4100. The [2Fe–2S] cluster of SoxR exhibited characteristic absorption peaks at 414 nm, 448 nm, and 548 nm. Upon the addition of increasing concentrations of Zn^2+^, these peaks showed a marked decrease, indicating that higher Zn^2+^ levels led to a more pronounced disruption of the SoxR [2Fe–2S] cluster. This pattern resembled the expression profile of SoxR in the *iscU*^*-*^*/iscA*^*-*^*/fdx*^*-*^ knockout strain. We further validated the specificity of zinc's effect on SoxR using electron paramagnetic resonance (EPR) spectroscopy. Control Fe–S cluster proteins, including FhuF (containing a [2Fe–2S] cluster), IlvD (containing a [4Fe–4S] cluster), and BioB (containing both [2Fe–2S] and [4Fe–4S] clusters), were analyzed, confirming that zinc specifically targeted SoxR. Additionally, we observed that the *soxS*^*-*^ mutant strain exhibited increased sensitivity to oxidative stress on standard LB agar plates compared to other strains. A well-characterized response of *E. coli* to oxidative stress is the activation of the *soxRS* regulon [45]. This activation induces the expression of over 100 genes, including the gene encoding manganese-containing superoxide dismutase (SOD) [46], a critical regulator of the bacterial oxidative stress response [47,48]. Upon *soxS* complementation, this phenotype was reversed, further supporting our proposed regulatory pathway. Moreover, we found that zinc in combination with an oxidative stress-inducing agent exacerbated bacterial sensitivity to oxidative stress. This finding suggests a potential strategy for targeting bacterial resistance mechanisms.

Zinc is an essential element for cellular growth and differentiation [49]. Its total intracellular concentration is typically maintained at submillimolar levels, primarily in a protein-bound form [40]. Previous studies have shown that a transient increase in intracellular zinc to nanomolar levels induces *zntA* transcription via ZntR, as part of the cellular detoxification response [49,50]. This suggests that *E. coli* can tolerate low levels of zinc. However, at higher concentrations, we demonstrated that zinc regulation in *E. coli* is not primarily mediated by ZntA. Our study revealed that under millimolar zinc stress, zinc specifically inhibits the assembly of the SoxR [2Fe–2S] cluster. This finding expands the traditional understanding of SoxR as a redox sensor for superoxide/NO [51,52], highlighting its novel role in zinc homeostasis regulation via metal toxicity sensing. At high concentrations, zinc disrupts the SoxR-SoxS signaling pathway, ultimately downregulating the expression of the zinc uptake transporter ZnuACB. This finding is consistent with the essential role of the ZnuACB system in zinc acquisition in enterotoxigenic *E. coli* (ETEC) [53]. The ZnuABC transport system is known to significantly enhance the ability of several pathogens to proliferate within infected hosts and cause disease [38,54]. For instance, ZnuABC is essential for the virulence of *Chromobacterium violaceum*, enabling the bacterium to overcome host-imposed zinc limitation and establish infection [55]. Similarly, a zinc acquisition system in *Acinetobacter baumannii* confers resistance to calprotectin-mediated zinc sequestration, facilitating bacterial persistence and pathogenesis [56]. As microbial competition with host cells for zinc is critical for establishing infections, our study provides novel insights into the role of this regulatory axis in bacterial zinc homeostasis, offering a new perspective on pathogenic infection mechanisms. Vitamin K plays a crucial role in enhancing membrane permeability and combating antimicrobial resistance (AMR), particularly by inhibiting the efflux of antimicrobial agents from bacterial cells [57]. The antibacterial properties of vitamin K_3_ have been documented [58]. Our study revealed that zinc, in combination with this superoxide-inducing agent, further sensitizes bacteria to oxidative stress, leading to growth inhibition at lower concentrations of the inducer.

While the zinc concentrations used in this study exceed typical physiological levels, they were intentionally chosen to model acute zinc overload conditions that bacteria may transiently encounter under host-imposed metal stress or during antimicrobial exposure. Host immune cells are known to manipulate metal ion availability as part of nutritional immunity, including the release of zinc and copper at infection sites to restrict microbial proliferation [59]. Under such conditions, *E. coli* and other pathogens may experience localized bursts of zinc stress comparable to the experimental conditions used here [60]. These stress-mimicking conditions enabled us to mechanistically dissect how excess zinc perturbs SoxR [2Fe–2S] cluster integrity and redox sensing, revealing a biochemical vulnerability that may underlie bacterial adaptation to metal toxicity [61]. It should be noted that, following zinc-induced Fe–S cluster disruption, SoxR may exist either as an apo-protein or potentially as a zinc-bound form, given the presence of multiple cysteine residues normally involved in cluster coordination. Although zinc exposure caused pronounced SoxR instability and precipitation in our experiments, this does not formally exclude the existence of a minor soluble Zn-bound fraction. Importantly, regardless of whether SoxR adopts an apo or Zn-bound state, both forms are expected to be transcriptionally inactive, leading to reduced soxS expression under zinc stress. It should also be noted that under severe zinc overload, SoxR precipitation is likely to occur *in vivo*, which may further contribute to functional inactivation of the regulator in addition to Fe–S cluster disruption.

The possible role of the ferric uptake regulator (Fur) should also be considered in the context of zinc–iron balance. Fur could be influenced by zinc ions, acting then in the all-regulatory network related to iron uptake, oxidative stress response and Fe–S biogenesis. However, the possibility of such an effect remains to be further confirmed.

In summary, this study demonstrates that zinc excess directly disassembles the SoxR [2Fe–2S] cluster—a pivotal event that dysregulates bacterial oxidative defense through the SoxR-SoxS-ZnuACB/SOD axis. By coupling zinc overload to compromised redox homeostasis, we uncover a mechanistic paradigm wherein Fe–S cluster integrity dictates bacterial oxidative stress adaptation. These findings redefine zinc's role as a disruptor of Fe–S redox sensors and provide a strategic framework for combating antibiotic resistance by targeting metal-induced oxidative vulnerability.

## 4 Materials and methods

### 4.1 Strains and plasmid construction

The wild-type *E*. *coli* MC4100 strain carrying no exogenous plasmids was obtained from the China General Microbiological Culture Collection Center (CGMCC). The empty pBAD vector and cold-shock expression vector pCold I were purchased from Takara Bio Inc. (Kusatsu, Japan). The genes encoding SoxS was amplified from *E*. *coli* by PCR and cloned into the expression plasmid pBAD. Each plasmid was introduced into *E. coli* MC4100 cells. Using a one step cloning kit (C112-01, Vazyme Biotech, Nanjing, China), we successfully constructed three cold-shock expression plasmids (pCold I-ZnuA, pCold I-ZnuB, pCold I-ZnuC) and two arabinose-inducible expression plasmids (pBAD-SoxR, pBAD-SoxS).

### 4.2 Protein expression and purification

The pBAD-BioB had been previously constructed [26], and pBAD-IlvD, pBAD-FhuF and pBAD-SoxR were available from earlier laboratory work [62]. The transformed cells were cultured to an optical density at 600 nm (OD_600_) of 0.6, and recombinant protein expression was induced by adding 20 % L-arab at a 1:5000 dilution for 4 h. The cells were harvested and washed twice with protein purification buffer (500 mM NaCl, 20 mM Tris, pH 8.0). The purity of the recombinant proteins was assessed by SDS-PAGE followed by Coomassie Brilliant Blue staining. The concentration of the purified proteins was determined by measuring absorbance at 280 nm using the published extinction coefficient.

### 4.3 Gene knockout in *E. coli* cells

The *soxR*, *soxS* and *znuACB* genes were deleted from the *E*. *coli* MC4100 wild-type strain using a previously established protocol developed in our laboratory [63]. The *suf*^*-*^ and *isc*^*-*^ mutant strains had been previously constructed [27], and *iscU*^−^/*iscA*^−^/*fdx*^−^ knockout strains were available from earlier laboratory work [26].

### 4.4 EPR measurements

Following culture collection, the wild-type *E*. *coli* strain harboring the pBAD-SoxR overexpression plasmid was adjusted to an optical density (OD600) of 100. Subsequently, zinc was added at graded concentrations for subsequent analyses. The EPR spectra were recorded at X-band on a Bruker ESP-300 spectrometer using an Oxford Instruments ESR-900 flow cryostat (Chemistry Department/LSU). The EPR conditions were: microwave frequency, 9.45 GHz; microwave power, 20 mW; modulation frequency, 100 kHz; modulation amplitude, 2.0 mT; sample temperature, 20 K; receive gain, 1.0 × 10^5^.

### 4.5 IlvD activity assay

The enzymatic activity of IlvD was determined using the substrate D,L-2,3-dihydroxy-isovalerate. A defined amount of Cell lysate of *E*. *coli* expressing IlvD was added to a pre-incubation mixture containing 50 mM Tris (pH 8.0), 10 mM MgCl_2_, and 10 mM D,L-2,3-dihydroxy-isovalerate, and the reaction was carried out at 37 °C. The accumulation of reaction products (oxo acids) over 1 min was monitored by measuring the absorbance change at 240 nm, using an extinction coefficient of 0.19 mM^−1^ cm^−1^. Each sample was analyzed in triplicate.

### 4.6 Metal content analyses

The total zinc content in cellular samples was determined using 4-(2-pyridylazo)-1,3-benzenediol (PAR). The iron content in protein samples was measured using the iron indicator FerroZine, following the method described in the literature [64]. The total zinc and iron content in *Escherichia coli* was also quantified using inductively coupled plasma mass spectrometry (ICP-MS). *E. coli* cultures grown overnight were diluted 1:100 into 50 mL of LB medium and incubated at 37 °C with shaking at 250 rpm until the optical density (OD) at 600 nm reached 0.6. The cells were harvested by centrifugation at 4000 rpm for 10 min and washed three times with DB buffer. The OD of the bacterial suspension was adjusted to 10. A 4 mL aliquot of the suspension was mixed with 4 mL of 35 % nitric acid. The mixture was subjected to microwave digestion, and the iron and zinc content was quantified using ICP-MS.

### 4.7 Plate growth assay

Single colonies were picked from an LB plate and inoculated into 3 mL of LB liquid medium for overnight growth. The bacterial cells were washed 2–3 times with saline and adjusted to an optical density at 600 nm (OD_600_) of 0.02. A 2 μL aliquot was dropped vertically onto an LB plate. The growth of *E. coli* was observed after 20 h.

### 4.8 Q-PCR

Total RNA was extracted using the FreeZol Reagent RNA Isolation Kit (R711-01, Vazyme Biotech, Nanjing, China) and reverse-transcribed into cDNA using the Reverse Transcription RT Kit (11141ES60, Yeasen Biotechnology, Shanghai, China). Gene-specific primers were used for real-time PCR amplification. PCR reactions were performed in triplicate, with *mdoG* as the internal reference gene [65]. The relative cDNA levels were determined using the comparative Ct method. qPCR primers were synthesized by GenScript Biotech, and their sequences are listed in Table 1.

**Table 1:** Q-PCR primer sequences.

Genes	F-sequences	R-sequences
*soxS*	TGACGCATCAGACGCTTGGC	AAATCGGACGCTCGGTGGT
*znuA*	CGCAATCGTTACCTGCTTCG	GCCCATCGGATGTAAAACGC
*znuB*	CTGGTGTCGCTGTTTTGGTG	TAACAGTGCCGCACATAGCA
*znuC*	ACTGCGTCGTGAACTGGATT	GAAACAACTTCCGGTGTGCC
*zntA*	TGCCCGTGTGCGTTAGTTAT	TAACACGACCCAGCTGTTCC

## CRediT authorship contribution statement

**Jie Feng:** Data curation, Formal analysis, Investigation, Validation, Visualization, Writing – original draft, Writing – review & editing. **Feng Liang:** Formal analysis, Investigation, Writing – review & editing. **Yongguang Zhou:** Data curation, Investigation, Visualization, Writing – review & editing. **Shihao Wen:** Investigation, Writing – review & editing. **Yue Chen:** Investigation, Writing – review & editing. **Binjie Ge:** Investigation, Writing – review & editing. **Wenjing Zhang:** Investigation, Writing – review & editing. **Jie Wang:** Writing – review & editing. **Runyu Chen:** Writing – review & editing. **Yin Zhang:** Writing – review & editing. **Jianghui Li:** Project administration, Supervision, Writing – review & editing. **Wu Wang:** Methodology, Supervision, Writing – review & editing. **Guoqiang Tan:** Conceptualization, Funding acquisition, Resources, Supervision, Writing – review & editing.

## Declaration of competing interest

The authors declare that they have no known competing financial interests or personal relationships that could have appeared to influence the work reported in this paper.

## Data Availability

Data will be made available on request.
