# Supplementary material for: Zinc overload disrupts SoxR [2Fe–2S] clusters to drive redox-metallic crosstalk via SoxS-ZnuACB in Escherichia coli
Source: Redox Biol. 2026 Jan 8;90:104013. doi: 10.1016/j.redox.2026.104013 (PMC12818246; doi:10.1016/j.redox.2026.104013)
Supplement: Multimedia component 1 [file mmc1.docx]

**
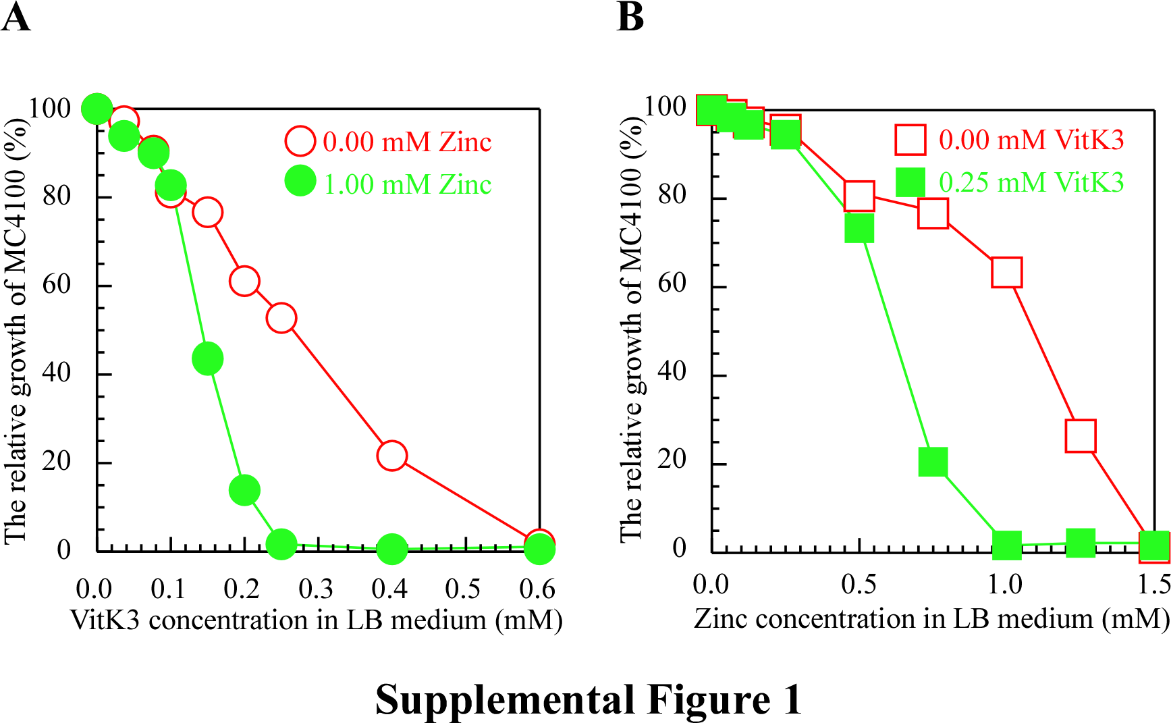
**

**Supplemental Figure 1: Relative bacterial growth under zinc and vitamin K3 stress.** Growth curves of wild-type *E. coli* MC4100 cells cultured under different concentrations of vitamin K3 with or without 1 mM Zinc (A), and under different concentrations of Zinc with or without 0.25 mM vitamin K3 (B).

**
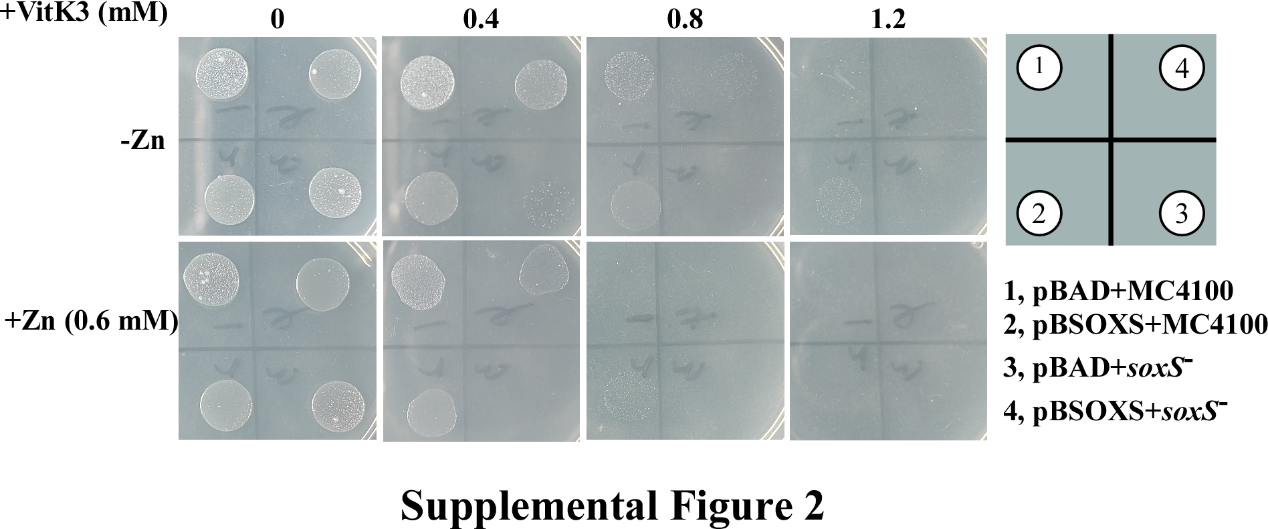
**

**Supplemental Figure 2: Complementation of *soxS* in *E. coli*.** Susceptibility analysis of SoxS-supplemented bacteria to oxidative stress. Under aerobic conditions, pBAD+MC4100 (1), pBSoxS+MC4100 (2), pBAD+*soxS*^-^ (3), and pBSoxS+ *soxS*^-^ (4) were inoculated at an OD₆₀₀ of 0.02 onto LB agar plates with or without 0.6 mM Zn²⁺ and different concentrations vitamin K₃ (VitK₃) as an oxidative stress inducer. Plates were incubated for 24 h.


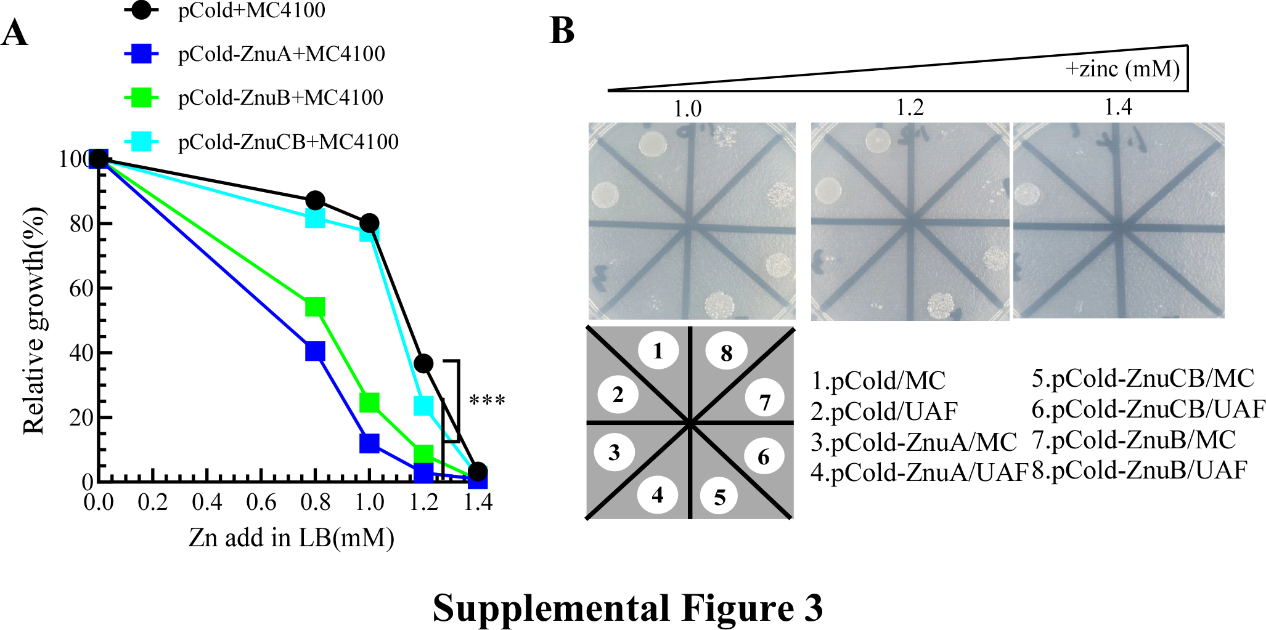


**Supplemental Figure 3:** (**A**). Growth curves of wild-type *E. coli* cells of MC4100 complemented with pCold-*znuA*, pCold-*znuCB*, or pCold-*znuB*, grown in LB medium supplemented with 0.0, 0.8, 1.0, 1.2, or 1.4 mM ZnSO₄. **(B).** Zinc sensitivity analysis of knockout strains. Under aerobic conditions, *E. coli* strains carrying different plasmid constructs—pCold/MC4100 (1), pCold/*iscU⁻/iscA⁻/fdx⁻* (2), pCold-ZnuA/MC4100 (3), pCold-ZnuA / *iscU⁻/iscA⁻/fdx⁻* (4), pCold-ZnuCB/MC4100 (5), pCold-ZnuCB/ *iscU⁻/iscA⁻/fdx⁻* (6), pCold-ZnuB/MC4100 (7) and pCold-ZnuB/ *iscU⁻/iscA⁻/fdx⁻* (8)—were inoculated on LB agar plates containing 0 mM, 1.4 mM, or 1.6 mM ZnSO₄ and incubated for 24 h.


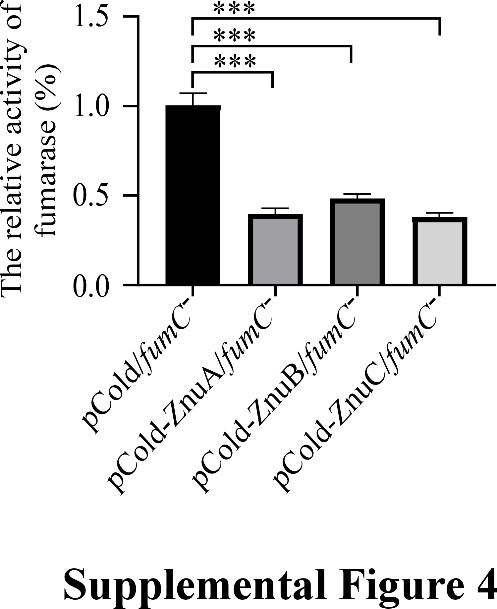


**Supplemental Figure 4: Changes in fumarase activity of *E. coli*.** Fumarase activity measured in *fumC⁻* strains carrying an empty vector and in *fumC⁻* strains overexpressing ZnuA, ZnuB, and ZnuC.
